# Applying journey mapping and human-centered design to improve critical care delivery for patients with acute respiratory failure

**DOI:** 10.1186/s12913-025-13864-6

**Published:** 2025-12-30

**Authors:** Sara E. Golden, Patrick G. Lyons, Allison Young, Scott Warner, Anais Tuepker, Ian Ilea, Donald R. Sullivan, Christopher G. Slatore, Kelly C. Vranas

**Affiliations:** 1 Center to Improve Veteran Involvement in Care, VA Portland Health Care, 3710 SW US Veterans Hospital Rd RD66, Portland, OR 97239 USA; 2 Division of Pulmonary, Allergy, & Critical Care Medicine, Oregon Health & Science University, 3181 SW Sam Jackson Park Rd, Portland, OR 97239 USA; 3 Division of Cardiovascular Medicine, University of Virginia, 1215 Lee St, Charlottesville, VA 22908 USA; 4 Department of Family Medicine, Oregon Health & Science University, 3181 SW Sam Jackson Park Rd, Portland, OR 97239 USA; 5 Section of Pulmonary & Critical Care Medicine, VA Portland Health Care System, 3710 SW US Veterans Hospital Rd RD66, Portland, OR 97239 USA

**Keywords:** Human-centered design, Triage, Intensive care units, Qualitative research, Respiratory insufficiency

## Abstract

**Background:**

Acute respiratory failure is a common cause for hospitalization and intensive care unit (ICU) admission. Prior literature has found that factors unrelated to patients’ illness severity or clinical needs contribute to substantial variability in ICU admission rates across hospitals. Overuse of the ICU for patients unlikely to benefit from critical care is inefficient, contributes to rising costs, and reduces access to critical care for those who most need it. As part of efforts to improve the efficiency and value of critical care, we utilized human-centered design to create a prototype, system-level intervention designed to optimize ICU utilization for patients hospitalized with acute respiratory failure.

**Methods:**

We created a multidisciplinary taskforce and conducted four meetings over a 5-month period in 2022 at a VA medical center. We used journey mapping to depict the care continuum of acute respiratory failure patients and identify facilitators/barriers to high-value care; next, we integrated qualitative methods using rapid team-based analysis with human-centered design to develop a system-level intervention to guide triage decisions and tailor care-delivery processes.

**Results:**

Our taskforce was composed of ten participants (including nurses/physicians/respiratory therapists) with clinical and leadership roles in the emergency department, medical/surgical wards, and ICU. We created a service blueprint map and leveraged it to identify themes influencing ICU utilization among patients with acute respiratory failure, including: (1) hospital organization and care processes (e.g., lack of established ICU admission criteria); (2) available resources outside the ICU (e.g., staffing/bed shortages); and (3) staff interactions (e.g., lack of communication/coordination between clinicians/departments). Informed by these results, the taskforce designed a prototype intervention with four components: (a) create explicit ICU admission criteria; (b) assign levels of care based on patients’ needs; (c) geographically cohort patients with shared needs outside the ICU; and (d) re-engineer rapid-response teams to proactively assess/follow patients outside the ICU.

**Conclusions:**

We combined qualitative and human-centered design methodologies to develop a prototype intervention designed to improve the value of care for patients with acute respiratory failure. Future studies will pilot test the feasibility and outcomes of the intervention we have developed in this study.

**Supplementary Information:**

The online version contains supplementary material available at 10.1186/s12913-025-13864-6.

## Introduction

Acute respiratory failure is a common cause of hospitalization and intensive care unit (ICU) admission [1]. Although acute respiratory failure can be associated with high morbidity and mortality, many patients with this syndrome are not critically ill and do not require ICU-level care [2]. Hospitals vary widely in how ICU admitting decisions are made for these patients, and little evidence exists to guide such decisions [3]. Because ICU beds are a limited, costly, and scarce resource, ICU admission for patients who are not critically ill is inefficient, costly, and decreases access to critical care for those who most need it [2, 4].

A key goal of optimizing critical care delivery and healthcare resource utilization is to identify and admit patients to the ICU who will truly benefit from such resource-intensive care [4, 5]. However, prior literature has found that hospital factors (e.g., hospital culture, practice patterns) unrelated to patients’ illness severity or clinical needs contribute significantly to variability in ICU admission rates [6]. These findings are of great policy relevance, as they suggest that comparable patients may be treated differently based solely on their location of treatment [6]. They also highlight existing opportunity to improve the quality and efficiency of our critical care delivery system through the development of hospital-level interventions that guide ICU admitting decisions for patients in a way that is tailored to different risk groups and sensitive to different health systems [6, 7]. 

While recent studies have attempted to quantify the benefits of ICU admission for patients with acute respiratory failure, [5, 8, 9] little is known about how to design or implement hospital-level interventions to guide triage of patients in the emergency department (ED). Therefore, we sought to utilize human-centered design – a research and innovation framework in which the needs, behaviors, and experiences of end-users drive product or service design and implementation – to develop a hospital-level intervention that guides ICU utilization for patients presenting to the ED with acute respiratory failure, with the goal of improving efficiency and quality of care for this patient population. We chose to focus on acute respiratory failure because: (a) it is one of the most common reasons for ICU admission worldwide, and (b) it can be caused by a variety of etiologies (e.g., pneumonia, chronic obstructive lung disease, etc.) for which ICU admission rates vary widely across hospitals without detectable impacts on outcomes or cost [9–12]. Here, we describe using qualitative methods to evaluate clinicians’ perspectives and journey mapping to identify specific barriers and facilitators to efficient, high-quality care across the continuum of inpatient care. We then utilize these qualitative findings to inform the development of a hospital-level intervention designed to guide the triage of patients with acute respiratory failure throughout the acute care setting using human-centered methodology.

## Methods

### Study design

**Fig. 1:**
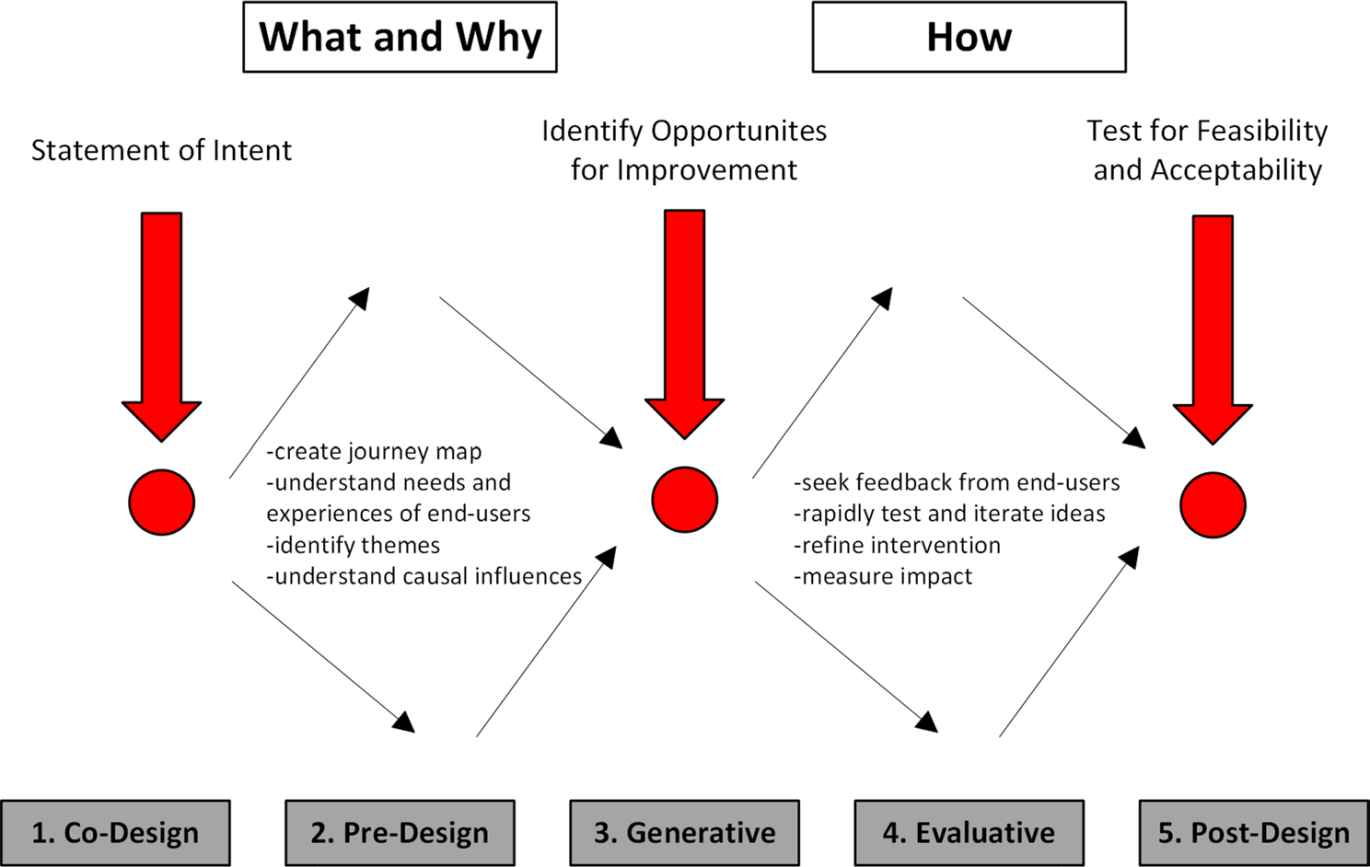
Overview of Study Design Utilizing Human-Centered Design Methodology* *adapted from Mastering design thinking. Sloan School of Management; 2023 [cited 2023 Jul 18]. Available from https://executive.mit.edu/course/mastering-design-thinking/a056g00000URaa4AAD.html

Human-centered design is a systematic method to optimize the design of products or interventions using an iterative approach, taking into account the contexts of use, local environment, user characteristic, and workflow [13]. Of note, it places strong emphasis on empathy for the end-user to help identify relevant problems and prioritize feasible, acceptable solutions [14]. For this study, we utilized a previously described methodological framework for human-centered design that includes four distinct phases: (1) Pre-design, in which feedback is sought from users about their experience using products and systems, and sensitizing to the problem to be addressed in the design process; (2) Generative, in which ideas, insights, and concepts are tested and refined with users in preparation for the development of prototype interventions; (3) Evaluative, in which prototypes are further developed and their effects and effectiveness tested with end-users; and (4) Post-design, which examines how users experience the design in practice in order to iterate in future design cycles based on identified needs and use patterns (Fig. 1) [13, 15]. Herein, we report the Pre-design and Generative phases of this framework, including qualitative data collected through design workshops with a multidisciplinary taskforce comprised of clinicians and hospital administrators.

As a first step in the human-centered design process, we created a journey map depicting the current-state triage processes for patients presenting to the ED with acute respiratory failure. Journey mapping – an emerging concept that has evolved out of the service design field – is increasingly being applied in healthcare to visually map the care continuum and identify inefficiencies in care processes [16–18]. For this study, we chose to create a service blueprint map, a specific form of journey map that describes a service experience from a systems view, typically in a chronological format [16]. In contrast to a patient journey map – which focuses on what patients experience when they experience clinical care – a service blueprint map goes deeper to illustrate the clinicians’ actions and organizational processes that may or may not be visible to the patient. Service blueprint maps typically contain several categories that illustrate the main components of the service being mapped out and how they may (or may not) interact with each other [19, 20]. Notably, with its focus on healthcare delivery rather than the patient experience, a service blueprint map represents the perspectives and experiences of clinical and administrative stakeholders, allowing for illustration of critical timepoints in patients’ movement through the acute care setting through an organizational lens.

### Setting and participants

This study was conducted at a Veterans Affairs (VA) Hospital that is designated as a Level 1a complexity level, the highest level of complexity for VA hospitals (defined by their ability to provide both general and specialty surgical services). Affiliated with an academic medical center, it has medical students and trainees in all patient care areas (including the ED, ward, and ICU). The hospital has a combined 24-bed medical/surgical/cardiac ICU, 122 acute care beds, and no intermediate care unit (i.e., a hospital unit that provides a level of care between intensive care and care on the general acute care ward) [21]. At this hospital, the use of invasive mechanical ventilation, non-invasive ventilation, and high-flow nasal cannula, is generally limited to the ICU. Patients requiring low-flow or moderate-flow oxygen therapy are typically admitted to the general ward. Early warning scores are not routinely used in real time to triage patients. Finally, there is a rapid response team available 24 h/day, 7 days/week that includes an ICU nurse, a medical resident/and or critical care fellow rotating through the ICU, and a respiratory therapist (RT). This team will see patients reactively when called for by the primary team if concerns exist about clinical deterioration.

In the Pre-Design phase of human-centered design methodology, we first created a stakeholder taskforce representing clinicians and hospital administrators across the continuum of inpatient care. To identify participants for the taskforce, we approached the facility’s ICU director for participation; we then employed snowball sampling to identify a multidisciplinary taskforce of physicians, nurses, and RTs from the ED, ICU, and medical/surgical wards. In total, we invited 11 clinicians (each with a concomitant administrative role in their respective clinical sections) to participate in the study, with 10 consenting to do so (Table 1).

**Table 1:** Self-reported characteristics of taskforce participants

Clinical Role	Gender	Hospital Location
Nurse #1	Male	Emergency Department
Nurse #2	Male	Emergency Department
Nurse #3	Male	Medical/Surgical Ward
Nurse #4	Female	Intensive Care Unit
Respiratory Therapist #1	Male	Hospital-wide
Respiratory Therapist #2	Male	Hospital-wide
Physician #1	Male	Emergency Department
Physician #2	Female	Medical/Surgical Ward
Physician #3	Male	Medical/Surgical Ward
Physician #4	Male	Intensive Care Unit

### Data collection

We conducted a series of four, one-hour virtual design workshops with taskforce members over a 5-month period in 2022. Three research team members, including a critical care physician and health services researcher (KCV), an experienced qualitative researcher and social scientist (SEG), and a research coordinator (AY) conducted the meetings and took field notes. Prior to the first meeting, we created a facilitation guide (Supplement 1) based on a conceptual model adapted from a study measuring hospital-level variation in ICU utilization [6]. All workshops lasted between 55 and 65 min and were video-recorded and transcribed verbatim by trained research staff. Between each design workshop, KCV, SEG, and AY performed directed qualitative content analysis to identify themes relevant to ICU triage processes and inform the development of a prototype hospital-level intervention as described below.

### Phase 1: Pre-design phase of human-centered design

Prior to the first workshop, we generated a preliminary service blueprint map depicting the continuum of care for patients who present to the ED with acute respiratory failure based on prior literature synthesized with clinical experience [22, 23]. Patients with acute respiratory failure were defined broadly as those requiring admission for treatment of an acute, primary respiratory issue (e.g., shortness of breath, hypoxemia, and/or hypercapnia), without specifying underlying etiologies. During the first workshop, taskforce members were shown the preliminary service blueprint map and asked to describe whether/how the map depicted their experiences caring for and triaging patients who present to the ED with acute respiratory failure. Taskforce members were asked to provide modifications to the service blueprint map to enable a more accurate depiction of their clinical workflows. After the first design workshop, the study team performed qualitative analyses of the group’s discussion as outlined below to help visualize and identify gaps in key healthcare processes; results from these analyses were used to revise and refine the service blueprint map prior to the second workshop and to inform the subsequent discussion of a future intervention.

During the second workshop, the service blueprint map was iteratively refined by taskforce members until consensus was reached. Taskforce members were then asked to identify barriers to and/or facilitators of efficient, high-quality care for patients hospitalized with acute respiratory failure, leveraging their deep contextual knowledge of clinical care and hospital workflows. After the second design workshop, the study team again performed qualitative analyses of the focus group’s discussion; results from these analyses were used to inform the following co-design of a prototype hospital-level intervention described below.

### Phase 2: Generative phase of human-centered design

During the third design workshop, informed by results from qualitative analyses during the Phase 1, taskforce members co-designed a prototype, system-level intervention to guide triage and care of patients presenting to the ED with acute respiratory failure. Following the third design workshop, study team members conducted semi-structured interviews with individual taskforce members to refine the intervention and elicit feedback from participants outside the group setting. The study team again performed qualitative analyses of semi-structured interviews between the third and fourth design workshops; results from these analyses were used to inform the subsequent refinement of the prototype hospital-level intervention described below. At the fourth design workshop, taskforce members iterated and finalized the intervention, and discussed strategies for implementation (to be reported in a future publication).

### Data analysis

As described above, KCV, SEG, and AY used inductive and deductive thematic approaches to perform directed qualitative content analysis of the transcripts and field notes taken during each design workshop and semi-structured interview. KCV and SEG read the first two transcripts to create a preliminary codebook. After discussion, we independently coded the same first two transcripts and met to compare and reach consensus. Next, AY read the first two transcripts to become familiar with the data and the codebook. AY and SEG coded the next two transcripts together while refining the codebook, after which point no other changes were made. We coded the remaining six transcripts independently, using Atlas.ti v9 for data organization and an audit trail and memos for organization and synthesis. Our sample contained sufficient information power (i.e., capacity of a sample to provide appropriate and rich data) given our relatively narrow study aim, the specific deep expertise of our participants, robust dialogue with participants, and rigorous analyses [24]. This study was approved by our Institutional Review Board (#4439).

## Results

**Fig. 2:**
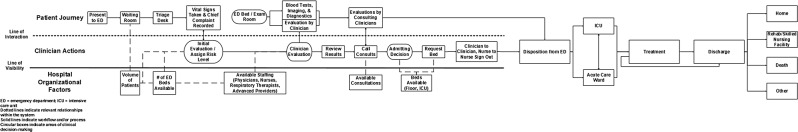
Service Blueprint Map of Current Acute Care Delivery for Adults Presenting with Acute Respiratory Failure. The map is divided into three rows, with the first row illustrating a patient presenting to the emergency department with acute respiratory failure’s journey through the acute care setting. The first and second rows are divided by the line of interaction that symbolizes the interplay between clinicians and patients that is more transparent. The second row depicts actions taken by clinicians in caring for these patients. The second and third rows are divided by the line of visibility that symbolizes the more hidden interplay between clinicians and the healthcare system. The third row illustrates hospital-level organizational factors that influence care delivery, but that may be less visible to patients and/or clinicians

The taskforce included four nurses, four physicians, and two RTs, with representation across the ED, general acute care wards, and the ICU (Table 1). The finalized service blueprint map of acute care delivery for adults presenting to the ED with acute respiratory failure is depicted in Fig. 2. This map represents the journey of a patient upon presentation to the emergency department, the actions taken by clinicians in caring for these patients, and additionally depicts the hospital-level organizational factors that impact these processes. Of note, the study team added clarifying labels in the final map.

### Qualitative analyses

**Fig. 3:**
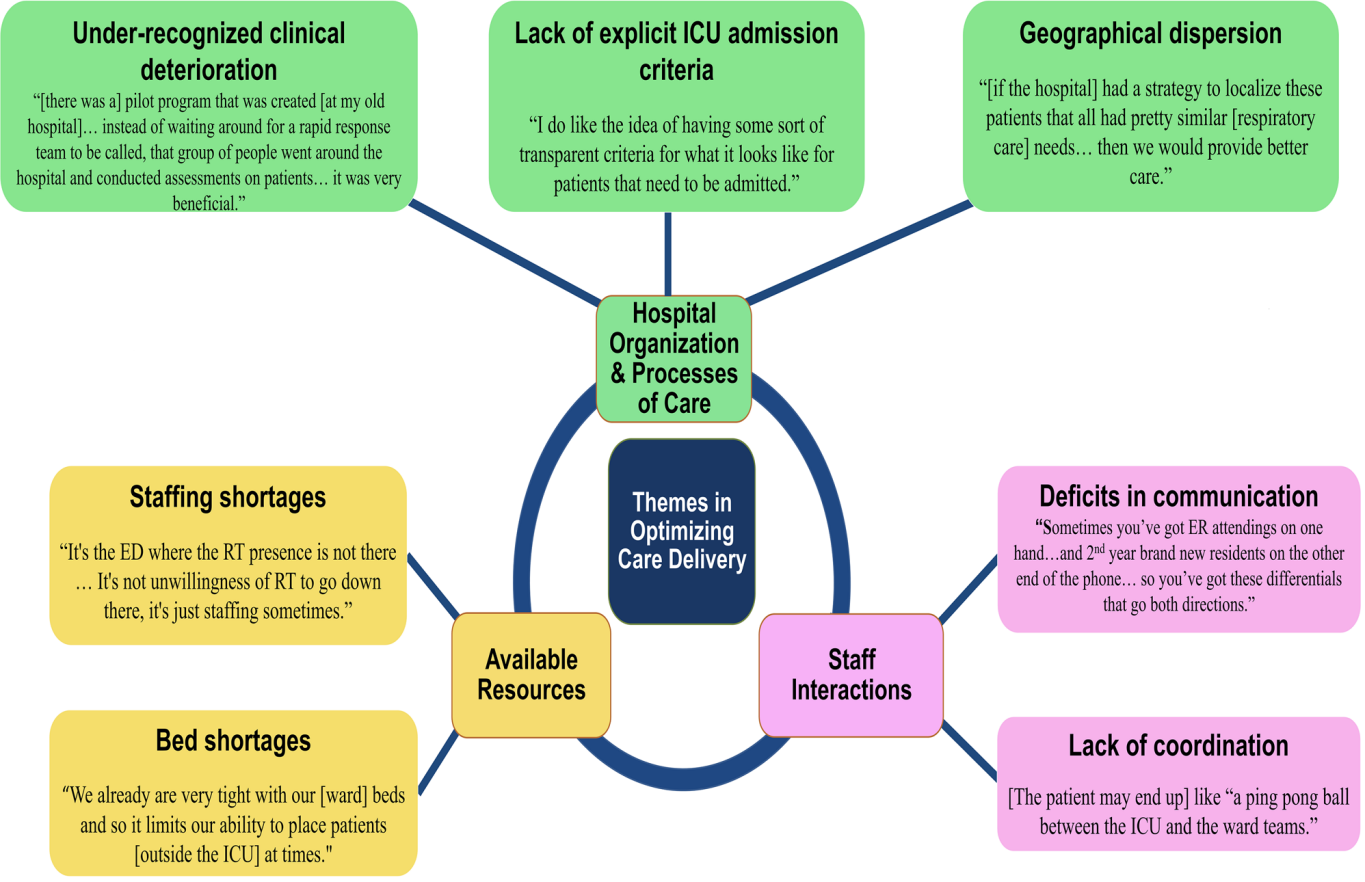
Themes and exemplary quotes describing factors influencing triage decisions and care delivery. This figure visually organizes our emerging themes and in combination with exemplary quotes serves to further describe factors influencing the triage and care of adults with acute respiratory failure at our VA hospital

In qualitative analyses of the design workshops, we identified three themes, each with several subthemes, encompassing factors that influence triage decisions and care delivery for patients hospitalized with acute respiratory failure. These include: (1) hospital organization and care processes; (2) available resources; and (3) communication and coordination between staff. Below, we describe each theme with representative quotations with additional quotes embedded in Fig. 3.

### Theme 1: Hospital organization and care processes

#### Lack of explicit ICU admission criteria leads to variability in ICU admitting decisions

Participants described how ICU admitting decisions from the ED varied depending on factors unrelated to patient needs (e.g., subjective clinician preference); they attributed much of this variation to the lack of explicit ICU admission criteria. Some participants were unsure as to whether specific ICU admission criteria existed, whereas others felt certain they did not. Participants noted that explicit admission criteria for patients with acute respiratory failure who did not require life-sustaining treatment would be helpful to standardize triage processes and avoid decisional conflicts and deliberation among clinicians regarding patients’ discharge dispositions. As one nurse stated “I do like the idea of having some sort of transparent criteria for what it looks like for patients that need to be admitted… It has always been kind of a push-pull [for] situations on the borderline.”

#### Geographic dispersion of patients with acute respiratory failure contributes to workflow inefficiencies

Participants described how hospitalized patients with acute respiratory failure outside the ICU were often geographically dispersed across the hospital, leading to workflow inefficiencies. In particular, RTs described wasting a lot of time “running around the hospital” to find patients—time that they felt could be better spent providing treatments and tailoring respiratory support interventions for their patients. Participants felt that geographic cohorting of patients with acute respiratory failure who were *not* critically ill outside the ICU would help create an “economy of scale” by concentrating the expertise and resources needed to care for these patients in a specific location. One RT noted that if the hospital “had a strategy to localize these patients that all had pretty similar [respiratory care] needs [outside of the ICU]… then we would provide better care.”

Furthermore, widespread consensus existed among participants regarding the potential benefit of an intermediate care unit as a specific example of geographic cohorting for “borderline” patients to improve care efficiency and outcomes. However, substantial uncertainty existed among participants regarding policies and procedures involving intermediate care units (e.g., requirements for physical proximity to the ICU, staffing models, which patients were appropriate for admission, etc.). Such administrative and logistical uncertainty has served as a substantial barrier to the creation of an intermediate care unit in the past and was perceived to be a substantial burden to future development of such a unit.

#### Concerns about inadequate detection of clinical deterioration among patients on the ward lead to unnecessary ICU admissions

Participants described concerns that clinical deterioration may go underrecognized on the ward as a common reason for ICU admission among patients who are not acutely critically ill. They raised concerns that currently-used rapid response teams designed to facilitate ICU transfer for unstable patients on the ward are often too reactive; several pointed out the potential benefits of shifting the role of rapid response teams to be more proactive in their assessments of “borderline” patients admitted outside the ICU.

### Domain 2: Available resources outside the ICU

#### Staffing and bed shortages on the ward leads to ICU overuse for non-critically ill patients

Inadequate staffing (particularly nurses and RTs) and bed availability on the wards was consistently mentioned as a barrier to the efficient and appropriate triage of patients with acute respiratory failure; participants described how this problem existed prior to the COVID-19 pandemic but had since been exacerbated by it. For example, participants noted how, due to staffing limitations, RTs are not stationed in the ED but are instead called there as needed. This staffing model often resulted in ED clinicians–who do not have specific expertise in applying or titrating devices such as high flow nasal cannula–managing patients with acute respiratory failure without the immediate assistance of RTs. For that reason, participants felt it would be beneficial to have a dedicated RT stationed in the ED to facilitate earlier assessment of patients with acute respiratory failure and provide more tailored therapies.

In addition, participants felt that bed shortages on the ward (often related to limited staffing) led to “overtriage” of patients to the ICU, when patients otherwise could have been managed on the ward with similar outcomes, but ended up in the ICU for reasons unrelated to their severity of illness or clinical needs. For example, one hospitalist said, “We already are very tight with our [ward] beds and so it limits our ability to place patients [outside the ICU] at times.”

### Theme 3: Staff interactions

#### Clinical silos, lack of communication, and power dynamics between clinicians contribute to delays in care

Many participants described how the care of patients with acute respiratory failure was influenced by interactions between clinicians, who often exist in geographic and clinical silos. Participants also felt that power dynamics (particularly in a teaching hospital) influenced the quality of care patients received. For example, one physician explained: “sometimes you’ve got [emergency medicine] attendings on one hand…and 2nd year brand new residents on the other end of the phone… so you’ve got these [power] differentials” that lead to conflict and delayed care. One physician explained how patients often end up “like a ping pong ball between the ICU and the ward teams.” Participants noted the value of face-to-face interactions between clinicians to improve communication and care coordination; they also noted the importance of early involvement of attending physicians when conflicts arise between clinical teams.

### Intervention prototype

**Fig. 4:**
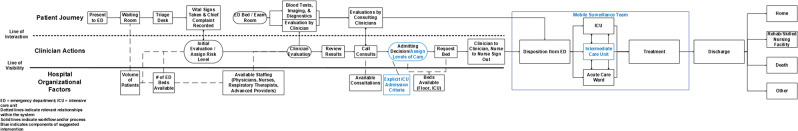
Service blueprint map of acute care delivery with overlay of potential intervention. This map shows acute care delivery at this hospital with the proposed intervention overlayed in blue. The map is divided into three rows, with the first row illustrating a patient presenting to the emergency department with acute respiratory failure’s journey through the acute care setting. The first and second rows are divided by the line of interaction that symbolizes the interplay between clinicians and patients that is more transparent. The second row depicts actions taken by clinicians in caring for these patients. The second and third rows are divided by the line of visibility that symbolizes the more hidden interplay between clinicians and the healthcare system. The third row illustrates hospital-level organizational factors that influence care delivery, but that may be less visible to patients and/or clinicians. The Intermediate Care Unit is one example of geographically cohorting patients with shared needs

The taskforce conceptualized four potential interventions that could be combined at a system-level depending on what is necessary and/or feasible within the context of a specific hospital environment as an outcome of the human-centered design process (Table 2; Fig. 4). Here, we briefly describe the intervention components that represent the output of human-centered design methodology. 

#### Component 1: Create Explicit ICU Admission Criteria

First, we propose the creation of standardized ICU admission criteria that are sensitive to the hospital-specific environment. Specifically, these criteria would define the need for ICU admission based on patients’ clinical needs (i.e., requiring life-sustaining treatment) at the conclusion of their evaluation in the ED (Table 2). Creating a culture of open dialogue and shared space between clinicians may also facilitate coordination and communication regarding admitting discussions.

**Table 2:** Potential intervention components example

Patients’ Needs	Level of Care	Location of Care	Example of Care Delivery Platform
Critically ill patients who need hourly monitoring or invasive life-sustaining therapies	1	ICU	N/A
Patients who need nursing interventions, lab work up, or monitoring every 2–4 h	2	Ward	Usual care PLUS scheduled evaluation by mobile surveillance team and effort to geographically cluster patients with shared needs, if possible
Stable patients who need testing or monitoring less frequently than q4h	3	Ward	Usual care

#### Component 2: Assign levels of care to patients based on clinical needs at time of admission

Complementary to component 1, we propose that patients presenting to the ED with acute respiratory failure be assigned a level of care based on objective criteria reflecting their clinical needs/acuity level. This classification could potentially help standardize triage processes by focusing on each patient’s specific monitoring and care requirements, [25] with the goal of reducing variability in ICU admitting decisions.

#### Component 3: Geographically cohort patients with shared needs

To help overcome staffing shortages and address existing workflow inefficiencies, we propose using a systems-engineering approach to geographically cohort patients with acute respiratory failure with similar clinical needs (e.g., frequent suctioning, respiratory treatments) but who are not critically ill in a specific ward location. Participants felt that cohorting patients in this way, with or without the creation of a dedicated “intermediate care unit,” could help create an economy of scale outside the ICU, streamlining workflows and maximizing efficiency of staffing resources, with the potential to improve patient outcomes.

#### Component 4: Re-engineer rapid response team to proactively follow patients outside the ICU

We propose shifting the paradigm for hospitals’ existing rapid response teams from being *reactive* to *proactive* [26, 27]. Specifically, this team (referred to as the “mobile surveillance team” by participants) could perform preemptive, scheduled evaluations of patients identified as “high-risk” for needing ICU transfer (according to assigned level of care as described in component 2) for a pre-set time period following admission.

#### Important considerations noted by participants

**Table 3:** Participant-reported benefits and concerns of each intervention component

Intervention component	Benefits	Concerns
Create Explicit ICU Admission Criteria	Aid in admission decisions and handoff communications Reduces variability in ICU admitting decisions	Difficulty evaluating criteria for complex patients and/or novice clinicians May be overly prescriptive
Assign Levels of Care to Patients Based on Clinical Needs at Time of Admission	Aid in triage decisions Standardize triage assignments	Difficult for more novice clinicians to evaluate needs
Geographically Cohort Patients with Shared Needs	Creates a space for respiratory therapists to be permanently located	Would increase acuity for nursing Requirement of additional training and monitoring of water
Re-engineer Rapid Response Team to Proactively Follow Patients Outside the ICU	Addition of support and monitoring	Difficulty staffing and managing

It is important to note both the potential benefits and downsides of such interventions as well as perceived by participants (Table 3). For example, while participants reported that standardizing ICU admission criteria would likely facilitate admitting decisions, it also risks being overly prescriptive (particularly in complex situations where clinical nuance exists). Similarly, cohorting patients was noted to be beneficial from the perspective of RTs (who generally work across multiple hospital units during a given shift and who often feel like they are “running all over the place”); on the other hand, nurses reported that cohorting patients with similar clinical needs risked increasing the acuity patients under the care of a given nurse. Finally, participants reported that having a more proactive Mobile Surveillance Team would help as an “additional layer of support to make everyone a little bit more comfortable” with having patients at a lower level of care, but leadership was concerned about “how to staff and manage such a team.” As participants noted, it will be important to consider unintended consequences of the intervention and prioritize its implementation in a systematic and transparent manner that reflects the culture and resources of a given hospital.

## Discussion

In this study, we created a multidisciplinary taskforce and created a service blueprint map as a first step in a human-centered design process to develop a prototype, system-level intervention with the goal of optimizing critical care delivery (including ICU admitting decisions) for patients presenting to the ED with acute respiratory failure [28]. Notably, although we focused on patients with acute respiratory failure because of its prevalence and the variability in ICU admission rates for this condition, it stands to reason that the methodology used in this study, as well as some of its conclusions, might be generalizable to other common medical conditions for which ICU care is typically provided but may not always be necessary. To our knowledge, this study is among the first to apply human-centered design methodology as part of efforts to improve the efficiency of our critical care delivery system. More broadly, this approach offers a potentially adaptable framework to systematically address existing complex problems within our healthcare delivery system (e.g., staffing shortages, inefficient use of resources) [29]. 

Our study adds to the literature in several ways. First, this study is unique in its application of human-centered design to develop system-level interventions designed to optimize critical care delivery for patients. While human-centered design is increasingly being used in the outpatient setting, [31–32] few studies report its use within the inpatient healthcare delivery system. One recent study utilized human-centered design to redesign handoffs between operating room and surgical ICU staff; [34] another applied human-centered design to design bar-coded medication administration among hospitalized patients [34]. Our study builds on this prior work by using human-centered design to create a system-level intervention that spans the continuum of inpatient care, taking into account how distinct departments and clinicians across specialties interact with each other to coordinate care for patients hospitalized with acute respiratory failure. Additionally, our use of human-centered design methodology enabled the study team to identify important communication barriers that exist across clinical silos, while simultaneously gaining buy-in of organizational leaders by incorporating them within the intervention development process.

Second, the creation of a service blueprint map in the context of healthcare delivery is relatively novel, [35] particularly across the continuum of inpatient care. Specifically, the service blueprint map enabled participants to visualize key “pain points” that interfere with the delivery of efficient care within a complex system and identify opportunities for improvement in these areas. In this way, service blueprint maps represent a useful tool within healthcare improvement initiatives since they allow participants to identify, reflect, and ideate on the interplay between user workflows, resource constraints, and physical spaces to create innovative solutions. Taken together, our methods and results provide a basis for future efforts to design and implement complex interventions within our inpatient care delivery system to improve efficiency and optimize the overall value of care.

Finally, we found that communication and coordination between clinicians in different specialties was perceived by study participants to substantially impact patient care. Prior literature has demonstrated that miscommunication between clinicians during transitions of care drives diagnostic error [36]. Our study builds on this by identifying how the lack of communication and collaboration between clinicians may negatively impact patient outcomes in the form of mis-triage of patients. Our findings suggest that such miscommunication may be, in part, mitigated by the creation of standardized ICU admitting processes and highlight the need for future research aimed at improving organizational culture and communication practices across clinical silos.

Our study also has limitations. First, the intervention has not yet been tested for feasibility, acceptability, or impact on outcomes. In future implementation studies, it will be important to examine the use of the intervention through audit and feedback methods, as well as its effect on outcomes such as ICU admission rates, hospital mortality, and rate of unplanned transfer to the ICU of patients admitted to the ward. Second, there were factors not discussed in detail during taskforce meetings that could be addressed in future implementation studies, including the use of early warning systems as part of the Mobile Surveillance team. Third, participants were from a single VA site, limiting generalizability; however, a benefit of human-centered design methodology is its ability to tailor solutions to meet end-users’ needs within a particular context. Furthermore, while the intervention itself may not be generalizable, the methods used to develop the intervention are broadly applicable to various settings. Additionally, because this study was focused on care processes and organizational factors influencing triage decisions and care delivery for patients with acute respiratory failure, we did not include patients in the human-centered design process; however, we recognize that patients’ perspectives are important to elicit and incorporate in future implementation studies.

## Conclusions

This study illustrates how human-centered design, combined with service blueprint mapping and rapid team-based qualitative analysis, can be utilized to derive insights from stakeholders across existing healthcare silos and design system-level interventions to improve the efficiency and value of critical care delivery. Future studies will pilot test the feasibility and outcomes of the intervention we have developed in this study.

## Supplementary Information

Below is the link to the electronic supplementary material.

Supplementary Material 1

## Data Availability

The datasets generated and/or analyzed during the current study are not publicly available to protect the privacy of our participants.

## Abbreviations

ED: Emergency Department
ICU: Intensive Care Unit
RT: Respiratory therapist
VA: Veterans Affairs
