# Supplementary material for: Applying journey mapping and human-centered design to improve critical care delivery for patients with acute respiratory failure
Source: BMC Health Serv Res. 2025 Dec 30;26:163. doi: 10.1186/s12913-025-13864-6 (PMC12860004; doi:10.1186/s12913-025-13864-6)
Supplement: Supplementary file 1 — Supplementary Material 1 [file 12913_2025_13864_MOESM1_ESM.pdf]

Taskforce Facilitation Guide

Journey map

- Walk through what happens when a patient with acute respiratory failure (ARF) presents to the emergency department (ED). How do they move through from the ED to the intensive care unit (ICU)? Are there any major components missing in this figure?
- Are there any existing protocols used to help decide which patients go to the ICU and which go to the floor?
  - Are there specific staffing ratios on floor versus ICU for physicians, registered nurses, and respiratory therapists?
- At what points do decisions need to be made? What influences those decisions?
  - Examples: patient-level (age, sex, race/ethnicity, socio-economic status, insurance, relationships), hospital-level (ICU bed supply, availability, occupancy; complexity level), provider-level (staffing model, teaching status, local culture)
- Probes:
  - How does the admission process work for patients who present to the ED?
  - What happens when the ICU or ward is full?
  - What happens when the ED is full?
  - How is the plan of care typically communicated between providers in the Emergency Department and the admitting team in the ICU or on the ward?
  - If there are no floor or ICU beds available for a patient in the ED, who manages the patient while they remain in the ED?
- From what you know, how much does this process change from one Veterans Affairs hospital to another? Or a Veteran Affairs hospital to other hospitals?

Needs assessment

- Evidence
  - What kind of information or evidence are you aware of that shows whether or not the intervention will work in your setting?
  - What evidence have you heard about from your own research? Practice guidelines? Published literature? Co-workers? Other settings?
  - What kind of supporting evidence or proof is needed about the effectiveness of the intervention to get staff on board?
- Communication
  - Can you describe your working relationships with your colleagues? Can you describe your working relationship with leaders?
  - How do you typically find out about new information, such as new initiatives, accomplishments, issues, When you need to get something done or to solve a problem, who are your "go-to" people?
- Culture
  - How would you describe the culture of your organization? Of your own setting or unit?
    - Do you feel like the culture of your own unit is different from the overall organization? In what ways?
  - How do you think your organization's culture (general beliefs, values, assumptions that people embrace) will affect the implementation of the intervention?
    - Can you describe an example that highlights this?
  - To what extent are new ideas embraced and used to make improvements in your organization? Can you describe a recent example?

- What is most frustrating about the process of trying to get patients the care they need in the hospital on a given day?
    - What would make those frustrations less?
- How have these frustrations changed with COVID?
- Has anything gotten better with COVID? If so, what?
- Include discussion on what has been implemented during COVID and felt to work well vs less well?
- What happens when there is disagreement between clinicians in the triage process?
- How helpful are early warning score systems? How do you use them?
- What is the role of a rapid response team?
- What is the role of a step-down unit?
- What are the major barriers to patient throughput in the hospital? How are these overcome?
    - Communication between consultants and primary team
    - Handoffs from one team to another
- How do we create resilience (the capacity to recover quickly from difficulties) within our healthcare system?
